# siRNA Therapeutics against Respiratory Viral Infections—What Have We Learned for Potential COVID‐19 Therapies?

**DOI:** 10.1002/adhm.202001650

**Published:** 2021-01-27

**Authors:** Aditi Mehta, Thomas Michler, Olivia M. Merkel

**Affiliations:** ^1^ Department of Pharmacy Pharmaceutical Technology and Biopharmaceutics Ludwig‐Maximilians‐Universität München Butenandtstraße 5 Munich 81377 Germany; ^2^ Institute of Virology Technische Universität München Trogerstr. 30 Munich 81675 Germany

**Keywords:** inhalation, nanomedicine, pulmonary delivery, respiratory virus, SARS‐CoV‐2, siRNA

## Abstract

Acute viral respiratory tract infections (AVRIs) are a major burden on human health and global economy and amongst the top five causes of death worldwide resulting in an estimated 3.9 million lives lost every year. In addition, new emerging respiratory viruses regularly cause outbreaks such as SARS‐CoV‐1 in 2003, the "Swine flu" in 2009, or most importantly the ongoing SARS‐CoV‐2 pandemic, which intensely impact global health, social life, and economy. Despite the prevalence of AVRIs and an urgent need, no vaccines—except for influenza—or effective treatments were available at the beginning of the COVID‐19 pandemic. However, the innate RNAi pathway offers the ability to develop nucleic acid‐based antiviral drugs. siRNA sequences against conserved, essential regions of the viral genome can prevent viral replication. In addition, viral infection can be averted prophylactically by silencing host genes essential for host–viral interactions. Unfortunately, delivering siRNAs to their target cells and intracellular site of action remains the principle hurdle toward their therapeutic use. Currently, siRNA formulations and chemical modifications are evaluated for their delivery. This progress report discusses the selection of antiviral siRNA sequences, delivery techniques to the infection sites, and provides an overview of antiviral siRNAs against respiratory viruses.

## Graphical abstract

Pulmonary delivery of siRNA holds great promise for inhibiting respiratory virus replication particularly when antiviral drugs and vaccines are not yet available. This progress report discusses previous attempts of silencing respiratory viruses by highlighting the choice of target gene regions, the opportunities for the field of siRNA delivery and challenges in the development of inhalable siRNA formulations, particularly against SARS‐CoV‐2.

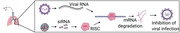

## 1 Introduction

Acute viral respiratory tract infections (AVRIs) are a major burden on human health and global economy. Even before the severe acute respiratory syndrome Coronavirus 2 (SARS‐CoV‐2) emerged in late 2019, AVRIs were amongst the top five causes of death worldwide resulting in an estimated 3.9 million lives lost every year. These numbers prompted the WHO to launch the "Battle against Respiratory Viruses" initiative.^[^ ^1^ ^]^ AVRIs furthermore have a huge economic impact being a leading cause for lost working days. The annual economic damage caused by influenza alone is estimated to reach 71 to 167 billion US dollars (USD).^[^ ^2^ ^]^

Importantly, while these numbers reflect the average annual burden of AVRIs, they do not account for regularly occurring outbreaks of new emerging viruses such as SARS‐CoV‐1 in 2003, the Influenza A strain California/7/2009 H1N1 (“Swine Flu”) in 2009, or most importantly the currently ongoing SARS‐CoV‐2 pandemic with a dramatic impact on global health, social life, politics and economy.

The majority of ARVIs are caused by Influenza viruses A and B, respiratory syncytial virus (RSV), parainfluenza viruses (PIV) 1–4, metapneumovirus (MPV), adenoviruses (AdV), coronaviruses (CoV), and rhinoviruses. The typical clinical pictures differ significantly between these viruses. Infections with highly pathogenic coronaviruses such as SARS‐CoV‐2 or the Middle East respiratory syndrome coronavirus (MERS‐CoV), but also influenza viruses, can trigger respiratory failure and significant lethality mainly in the elderly or patients with cardiovascular or respiratory disorders.^[^ ^3^ ^]^ In contrast, RSV impacts mainly small children causing obstructive bronchiolitis, clinically resembling bronchial asthma. Another risk group are immunocompromised individuals, especially stem cell transplanted patients, for whom not only SARS‐CoV‐2 or influenza infection poses a life‐threating risk, but also infections with RSV, PIV, MPV, or AdV. In contrast, so called "endemic" human coronaviruses (HCoV), which include HCoV‐NL63, HCoV‐229E, HCoV‐OC43, and HCoV‐HKU1, as well as rhinoviruses are not associated with significant lethality but mostly cause symptoms of the common cold. Last but not least, while not being primarily respiratory viruses, acute infection with measle virus or varicella zoster virus (VZV), as well as reactivation of latently persisting herpes viruses, including VZV, herpes simplex virus and cytomegalovirus, can cause severe pneumonia.

While most respiratory viruses cause pathology directly by a cytopathic effect or by triggering immune responses, a main complication of ARVIs is furthermore their ability to facilitate secondary bacterial infections, thereby provoking pneumonia, otitis, meningitis, and other acute inflammatory pathologies. In fact, ARVIs are estimated to be the primary reason for about half of the community‐acquired pneumonia cases,^[^ ^4^ ^]^ thus also impacting on antibiotic use and occurrence of multidrug resistant bacteria.

Given that the majority of respiratory virus infections does not lead to immunity after natural infection, effective vaccination for the majority of respiratory viruses appears desirable but difficult to be achieved.^[^ ^5^ ^]^

Although antiviral therapy is available for influenza, and with the recent approval of Remdesivir also for the treatment of SARS‐CoV‐2,^[^ ^6^ ^]^ these therapies are insufficient as they only have a limited effect on symptomatic disease, and lethality remains high.^[^ ^7^ ^]^ Ribavirin is sometimes used against severe RSV infections, however, has a questionable efficacy and unfavorable side effects. No antiviral therapies are available for the remaining respiratory viruses. As a result, the vast majority of ARVIs is currently being treated only symptomatically.

Taken together, developing effective antiviral therapies for respiratory viruses is an urgent unmet medical need. Such therapeutic intervention is expected to impact on disease symptoms and lethality, on the development of multi‐drug resistant (MDR) bacteria, and on the economy at large. Development of antiviral therapies is complicated by the fact that viruses to a large extent use cellular components for their replication. Thus, it is difficult to identify drugs which inhibit viral replication but do not interfere with physiological functions. Also, viruses have adapted in very specific ways showing a large variety of ways of propagation. As a result, no broad‐spectrum antiviral drugs are available as for the treatment of bacterial diseases. One strategy, however, which could allow to inhibit the replication of virtually any virus is to use RNA interference (RNAi). As the lung is relatively easily accessible for topical administration, for example, by inhalation, RNAi therapy appears to be an attractive approach to tackle respiratory viruses.^[^ ^8^ ^]^ While the effects of siRNA therapy are temporary, however, requiring multiple administrations, mRNA‐based vaccination approaches, in contrast, can in principle mediate immunity. On the other hand, reports about re‐infection with SARS‐CoV‐2^[^ ^9^ ^]^ and about quickly decreasing antibody titers in convalescent COVID‐19 patients are surfacing.^[^ ^10^ ^]^ Therefore, the longevity of immunization with an mRNA vaccine is not yet clear. Additionally, it has been suspected that SARS‐CoV‐2 may employ antibody‐dependent enhancement (ADE) to infect immune cells, an effect leading to more severe symptoms upon re‐infectious with the virus.^[^ ^5^ ^]^ Therefore, should vaccines not efficiently end this pandemic, we will need specific and effective therapeutics.

## 2 RNA Interference (RNAi)

**Figure 1:**
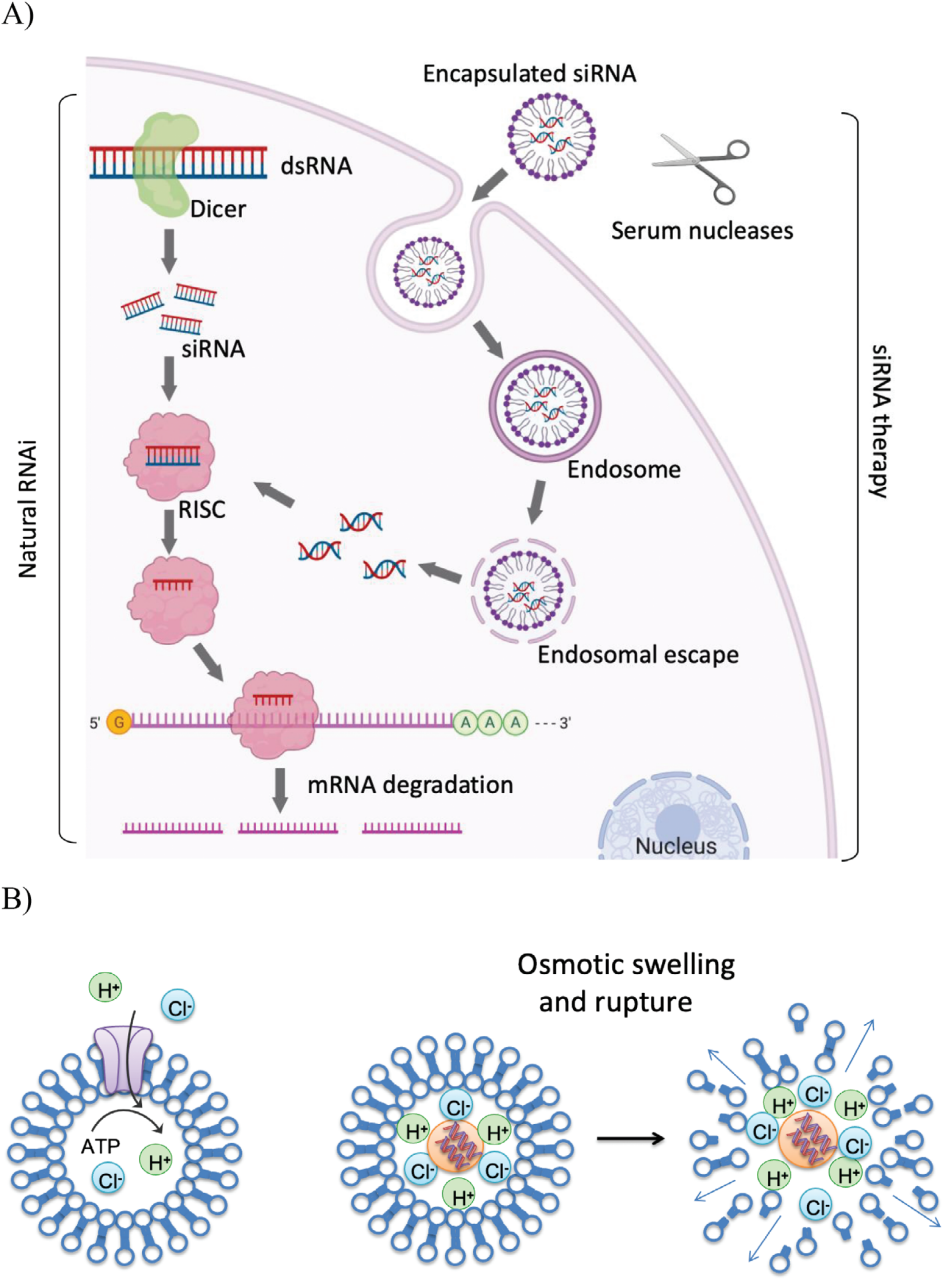
A) The mechanism of the RNA interference pathway. Double‐stranded RNA is cleaved by Dicer into short interfering RNAs. After incorporation into the RISC, the siRNA recognizes base‐complementary mRNA and guides its cleavage. When artificially applied via nanoparticles, siRNAs are taken up by the cells where they enter the early endosomes and following endosomal escape, siRNAs are released into the cytoplasm where they are taken up into the RISC. Figure created with Biorender.com. B) Endosomal escape of polyamine nanocarriers via the hypothesized proton sponge effect. Protons are pumped into the endosomes during endosomal ripening. Due to the buffering capacity of the polyamine, the proton flux increases and chloride counterions enter the endosomal compartment. This increased osmotic pressure leads to additional influx of water and the eventual burst of the endosome releasing the nanocarrier.

Since its discovery in 1998, RNAi has rapidly become one of the most exciting new areas of therapeutic drug development. RNAi is a conserved post transcriptional gene silencing pathway that regulates gene expression. During RNAi, small complementary non‐coding RNA species modulate messenger RNA (mRNA) stability and translation. The most widely used RNAi mediators are small interfering RNAs (siRNAs), that protect plants, fungi, and invertebrates via antiviral immune responses.^[^ ^11^ ^]^ The endogenous RNAi pathway occurs in two phases: during the first, the so‐called initiation phase, long dsRNA is cleaved by the endoribonuclease Dicer into siRNAs. The latter are short, double‐stranded dsRNAs (21–23 bp) with a characteristic structure of 19 nucleotides complementary to the target mRNA and of two nucleotide terminal 3′ overhangs (19 + 2mer structure).^[^ ^12^ ^]^ Subsequently the siRNA strands unwind, and in the second, or the effector phase, the guide or the antisense strand of the siRNAs is loaded into the multiprotein RNA‐induced silencing complex (RISC) which further guides the RISC to recognize and cleave the target transcript (i.e., silencing activity). This step is catalyzed by the AGO2 protein^[^ ^12^, ^13^ ^]^ (**Figure** **1**).

Whether RNAi plays a role in natural mammalian viral defense mechanisms still remains controversial.^[^ ^14^ ^]^ However all mammalian cells contain the evolutionarily conserved and required RNAi protein machinery, which therefore can be harnessed to inhibit mRNA expression using exogenously applied siRNA. Based on the sequence specificity of the siRNA to its target mRNA, RNAi allows the silencing of virtually any mRNA, whether endogenously occurring or a product of viral infections. In mammalian cells, introduction of chemically or enzymatically synthesized siRNAs which resemble the short and sticky overhang dsRNA Dicer cleavage products can also initiate RNAi,^[^ ^15^ ^]^ by‐passing the first phase (Figure 1). The first antiviral in vivo application of siRNAs was attempted in 2003 by targeting *Fas* (also known as *Tnfrsf6*) mRNA, encoding the Fas receptor in a mouse model of autoimmune hepatitis. Intravenous injection of *Fas siRNA* protected mice from liver failure and fibrosis and reduced Fas mRNA and protein in mouse hepatocytes for 10 days.^[^ ^16^ ^]^

RNAi has opened the doors to a new wave of therapies, unlocking molecular targets previously considered inaccessible. Over the last decade, multiple improvements have been applied to the archetypal siRNA design to improve the silencing efficiency, target recognition, and reduce toxicity and immunogenicity. These include the use of Dicer substrate siRNAs,^[^ ^17^ ^]^ small internally segmented siRNAs^[^ ^18^ ^]^, chemically modified siRNAs,^[^ ^19^ ^]^ and self‐delivering siRNAs which are both asymmetric and hydrophobic.^[^ ^20^ ^]^ In August 2018, in a landmark decision, the US Food and Drug Administration (FDA) approved the first siRNA‐based drug, patisiran (Alnylam Therapeutics) to treat polyneuropathy in patients with hereditary transthyretin‐mediated amyloidosis^[^ ^21^ ^]^ marking a new era for the very rapidly growing field of RNAi therapeutics. As of today, four siRNAs therapeutics have been approved by the FDA, namely patisiran (delivered via lipid nanoparticles),^[^ ^22^ ^]^ givosiran, lumasiran, and inclisiran (siRNAs that are chemically conjugated to GalNAc).^[^ ^23^ ^]^

## 3 RNAi Targeting Human Respiratory Viral Infections

As described above, antiviral therapies for respiratory viruses are urgently needed. Conventional systemic therapy using small molecules is available only for influenza, and to some extend also for RSV and SARS‐CoV‐2. Additionally, a humanized IgG1 antibody targeting the RSV fusion protein is approved as prophylaxis in neonates, but is not suitable to treat existing infection. The existing drugs target different steps of the viral replication cycles. The first event that can potentially be inhibited is viral attachment and entry into the target cells using Palivizumab (against RSV) or DAS181 (against influenza/parainfluenza) as therapeutics. Subsequent steps that can be targeted within the host cells involve the uncoating of the virus using M2 inhibitors (against Influenza), nucleic acid synthesis which can be blocked with ribavirin (against RSV), or the subsequent release of viral particles which is inhibited by Neuraminidase inhibitors (against influenza).^[^ ^24^ ^]^ However, possible cross reactivity with host proteins can cause adverse and severe side effects by inhibiting essential host cell functions. In contrast, RNAi catalytically targets the pathway upstream, modulating protein synthesis as opposed to its function. Moreover, the identification of the structure of the viral proteins and developing their small molecule inhibitors is an extremely time and resource intensive endeavor. On the other hand, RNAi‐based approaches offer a platform approach and can be used to target different infections by using a combination of different siRNAs or by interchanging siRNAs. Initial studies evaluated the effectiveness of siRNAs as antiviral agents in cell culture. The first report of successful siRNA‐mediated antiviral activity in cell culture was against RSV, a non‐segmented negative‐stranded RNA virus which causes bronchiolitis and pneumonia.^[^ ^25^ ^]^

RNAi is a particularly promising strategy to target viral infections against which neither effective vaccines nor specific therapeutics are yet available.^[^ ^5^ ^]^ While a vaccine against SARS‐CoV‐2 has been approved in several countries in the meantime, RNAi‐based antivirals are especially relevant for emerging viruses, as we experienced particularly in the early phase of the 2019/20 SARS‐CoV‐2 pandemic.

To harness the therapeutic potential of the natural RNAi pathway for potent and specific gene knockdown, siRNA formulations have to be optimized to avoid side effects of siRNA‐based drugs, the degradation of the siRNAs in the extracellular environment in vivo and enable endosomal escape of the siRNAs. Systemically injected nucleic acids face several hurdles including extracellular degradation by nucleases, renal clearance, interaction, and binding to plasma proteins and removal by the reticuloendothelial system. Subsequently, they must cross the cellular barriers and navigate the plasma membrane, escaping the endolysosomal and lysosomal degradation and must reach their intracellular site of action.^[^ ^26^ ^]^ For targeting respiratory viruses however, one can bypass systemic delivery focusing on direct, local delivery via the nasal route, that is, intranasal, or pulmonary delivery. Nonetheless, the siRNA still faces several of the mentioned obstacles and requires efficient formulation for inhalation administration.^[^ ^27^ ^]^

Many of the above‐mentioned challenges can be addressed by modulating the sequence,^[^ ^28^ ^]^ structural motifs, chemical modification of the nucleotides^[^ ^17^ ^]^ and engineering delivery formulations and routes.^[^ ^29^ ^]^ Side effects of RNAi based drugs remains a major concern and can result from multiple factors, including off target effects,^[^ ^30^ ^]^ immune response to RNAi triggered by innate sensors of foreign double‐stranded RNA (dsRNA),^[^ ^31^ ^]^ unintended RNAi activity in non‐target tissues and toxicity arising from excipients in the formulation^[^ ^32^ ^]^ and/or its byproducts after metabolism. In this progress report, we will therefore focus on the design of siRNA sequences, including the choice of the target genomic regions and suitable delivery strategies.

### 3.1 Design of siRNA—Genomic Target Region Selection

The majority of respiratory viruses have an RNA genome (including Influenza viruses, PIV, MPV, rhinoviruses, and corona viruses, but not AdV) and replicate using RdRP. Several studies showed that synthetic siRNAs are effective in specifically silencing viral RNAs, including RNA viruses which replicate exclusively in the cytoplasm. One exception is the influenza virus which uses cellular splicing factors in the nucleus. Unfortunately, the viral RNA dependent RNA polymerases (RdRP) lacks the proofreading ability of DNA polymerases which results in a high mutation rate of these viruses. For instance, the influenza A virus, a single stranded RNA virus, has an estimate of 2.3 × 10^−5^ substitutions per nucleotide per cell infection (µ_s/n/c_), whereas herpes simplex virus 1 (HSV‐1), a typical dsDNA virus, has a mutation rate of 5.9 × 10^−8^ µ_s/n/c_.^[^ ^33^ ^]^ The accurate and reliable estimation of the mutation rate of viruses is essential to understand their evolution and to develop strategies to combat them. This extreme variability and rapid evolution can allow viruses to cross the species barrier and cause severe outbreaks of previously unknown viruses resulting in either contained epidemics or, as we experience currently, worldwide pandemics. Also, the fast evolution enables viruses of the same species to escape protective immunity and lead to reinfections, as seen with most seasonally re‐occurring viruses. It furthermore poses discouraging challenges toward the design of effective vaccines and therapeutics against diseases caused by rapidly evolving RNA viruses.Despite the high mutation rate, most viruses possess genomic regions which are less likely to tolerate mutations as they could lead to reduced viral virulence. These include genes encoding for proteins essential for virus‐host interactions (for instance, spike proteins), viral replication or transcription (e.g., RdRP), and/or structurally‐conserved, non‐coding regions of the viral genome^[^ ^34^ ^]^. Targeting such regions alone, or several at a time, appears to be a promising strategy to tackle viral infections. Recent studies have shown that siRNAs suppressed gene expression and consequently inhibited the replication of SARS‐CoV, a novel, zoonotic positive RNA strand virus, in cultured cells by targeting the RdRP,^[^ ^35^ ^]^ the spike protein^[^ ^36^ ^]^ or the replicase 1A region of the genome.^[^ ^37^ ^]^ SARS was the first severe new disease to emerge in the 21st century. Multiple groups worked on siRNA therapeutics against SARS coronaviruses targeting the viral spike protein, the RdRP or the envelop protein genes in cell culture.^[^ ^36^, ^37^, ^38^ ^]^ In contrast, we recently identified ORF1 as a promising target in the genome of SARS‐CoV‐2,^[^ ^39^ ^]^ another novel, zoonotic positive RNA virus currently causing a world‐wide pandemic. For RSV, siRNAs targeting the viral fusion protein (F) and phosphoprotein (P), an essential subunit of the viral RNA dependent RNA polymerase^[^ ^25a^ ^]^ was shown to effectively inhibit RSV gene expression and growth in cell culture.^[^ ^25^ ^]^ Similarly, in parainfluenza virus (PIV) models, siRNA mediated knockdown of hemagglutinin‐neuraminidase and fusion proteins caused abrogation of virus mediated cell fusion.^[^ ^40^ ^]^ Another major public health concern is the seasonal flu, caused by various strains of the influenza virus. The latter is a negative strand RNA virus with a segmented genome belonging to the Orthomyxoviridae family. In 2006, Zhang et al. used siRNAs targeting host proteins to inhibit influenza virus infection in human 293T cells. siRNAs targeting the Ran‐binding protein 5, which is crucial for the nuclear import and assembly of the viral RdRP, resulted in delayed accumulation of viral RNAs in infected cells.^[^ ^41^ ^]^ Adenoviruses, in contrast, are non‐enveloped double‐stranded DNA viruses and cause widespread respiratory tract infections. siRNAs targeting adenoviral E1A mRNA precented viral replication, indicating a potential therapeutic effect against adenoviral infections.^[^ ^42^ ^]^ Off target RNAi activity can be minimized while designing the siRNAs using bioinformatic tools, such as BLAST to identify and eventually avoid homologous sequences where the 19 nt siRNA seed region can bind to unintentional mRNAs.^[^ ^28^ ^]^ However, despite extensive in silico efforts, unintended siRNA activity is often still observed in vitro and in vivo, and extensive pre‐clinical testing of siRNAs in a variety of in vitro, ex vivo, and in vivo models with complex genomes is critical. Unfortunately, despite vast testing, occasionally off‐target activity cannot be avoided. It has been shown that reducing the administered dose of siRNAs can help abrogate unintended mRNA cleavage. Since the RNAi pathway is a catalytic pathway and one single siRNA molecule can therefore bind to and regulate multiple mRNA copies, it allows for reducing the administered dose and thereby also allows to reduce off‐target activity associated toxicity.^[^ ^43^ ^]^

### 3.2 Chemical Modifications of siRNA

Another major point of concern are immunogenic reactions toward double stranded (ds) RNAs. Numerous dsRNA‐binding proteins can recognize dsRNA, such as the cytosolic dsRNA‐dependent protein kinase (PKR), pattern recognition receptors (PRRs), retinoic acid‐inducible gene I (RIG‐I)‐like receptors (RLRs) and the endosomal receptors, Toll like receptor (TLR) 3, 7, 8, and 9.^[^ ^31^, ^44^ ^]^ Once extracellular dsRNA is endocytosed, it is recognized by TLRs which signal from the endosomal membrane, whereas RLRs detect dsRNA in the cytoplasm and activate immunostimulatory pathways or apoptosis using a mitochondrial adaptor protein. These signaling pathways activate specific transcription factors and various proinflammatory as well as antiviral genes.^[^ ^45^ ^]^ This immune response has been a major problem for first‐generation synthetic siRNA drugs,^[^ ^46^ ^]^ and further investigation into the natural self/non‐self nucleic acid recognition have led to the identification of specific nucleotide modifications as a method to evade TLR activation.^[^ ^47^ ^]^

Chemical modifications of siRNAs are one of the most efficient approaches to enhance their stability, avoid recognition, and immunogenicity, and to improve efficiency of delivery. Both the ribose backbone and the nucleobases can each be modified to improve biopharmaceutical and therapeutic aspects of siRNA such as pharmacokinetics, pharmacodynamics, and biodistribution.^[^ ^48^ ^]^ Backbone modifications for siRNA include phosphodiester modifications to the groups that link consecutive ribose nucleosides. Interestingly, substituting the phosphodiester group with a phosphotriester facilitates cellular uptake of siRNAs and retention on serum components by eliminating their negative charge,^[^ ^49^ ^]^ enhancing their in vivo half‐life in circulation and improving in vivo delivery. Unfortunately, backbone modifications can also influence siRNA recognition by the RISC, and therefore sugar modifications are often preferred. Sugar modifications include replacing the 2′‐hydroxyl group of the ribose sugar with less nucleophilic groups such as 2′‐O‐methyl, 2′‐O‐methoxyethyl, and 2′‐fluoro modifications. However, using too many sugar modifications can influence the dissociation of the siRNAs during RISC loading. Immune cells distinguish foreign RNAs from “self” RNAs due to naturally occurring chemical modifications,^[^ ^50^ ^]^ which include sugar modifications such as 2′‐O‐methyl and base substitution with modified bases such as pseudouridine, 5′‐methylcytidine, N6‐methyladenosine, inosine, and N7‐methylguanosine. Thus, modifying siRNAs accordingly can allow them to escape immune detection^[^ ^51^ ^]^ to ultimately increase their silencing potential. The advantages and disadvantages of individual modifications have been reviewed extensively in the literature,^[^ ^52^ ^]^ and **Table** **2** summarizes some of the most frequently used RNA modifications. Recently, lipid conjugation to the 5′ and 3′ termini of siRNAs has gained popularity to improve their in vivo bioavailability by allowing them to associate with serum lipoproteins. Such lipid modifications include cholesterol, vitamin E, and others.

In 2008, Robbins and colleagues demonstrated that the immunostimulatory activity of siRNAs can potentially result in non‐specific therapeutic effects, which are especially relevant for antiviral therapies.^[^ ^53^ ^]^ Specifically, it was shown that the anti‐influenza effect observed in a mouse model resulted primarily from immune‐stimulatory effects of the active siRNA duplexes as compared to the control siRNA (siGFP) but was not a result of therapeutic RNAi. The authors reported that the control siRNA (siGFP) used in several publications had an exceptionally low immunostimulatory activity in comparison to the active anti‐influenza siRNA, resulting in a misinterpretation of the therapeutic RNAi effects. They therefore concluded that particularly in antiviral siRNA approaches, siRNA mediated immune activation needs to be monitored closely. The activation of the innate immune response and the production of interferons (IFNs) are well known to modulate viral replication. Thus, it is possible that the reduction of viral titer after treatment with the active siRNA was mediated by siRNA‐mediated IFN induction rather than an RNAi effect. Human peripheral blood mononuclear cells (PBMCs) from healthy donors were treated with lipid encapsulated or PEI‐polyplexed siRNA, and 24 h later IFN alpha (IFN*α*) levels were measured. Similarly, mice were administered the siRNA intravenously via tail vein injection, and IFN*α* levels were measured 6 h after treatment in blood collected after cardiac puncture. The authors showed that the control siRNA sequence used in several studies, a duplex targeting the green fluorescent protein (siGFP), had relatively low immunostimulatory effects in comparison with the specifically anti‐viral siRNA sequences, (e.g., siInf), both in vitro and in vivo.^[^ ^53^ ^]^

Such differences in the immunostimulatory potential of control versus active siRNAs can result in misinterpretation of the therapeutic efficacy caused by the siRNAs in animal models and must be fully characterized for both sequences. It has been shown that the incorporation of base modifications such m5C, m5U, s2U, m6A, pseudouridine, or extensive 2ʹ‐O‐methyl modifications at the ribose sugars in the more recent siRNAs can largely avoid immune recognition.^[^ ^54^ ^]^

### 3.3 Pulmonary Delivery of siRNA against Respiratory Viruses in the Literature

A problem rarely discussed in regard to siRNA side effects is that systemically administered RNA can accumulate in off‐target tissues. For developing therapeutic options using siRNAs against respiratory viruses, this problem is largely mitigated by using i) specific gene sequences in the viral genome as target regions, which have little to no homologous regions within the human genome and thereby display limited activity in non‐target tissues, and ii) by local delivery approaches such as intranasal or oral inhalation delivery.^[^ ^27^, ^55^ ^]^ In contrast to systemic delivery, localized pulmonary delivery allows drug access both to all regions of the respiratory system, owing to its large surface area, and systemic bioavailability mediated by the lung's rich blood supply.^[^ ^56^ ^]^ Other advantages are rapid onset of drug action and prolonged delivery periods as well as by‐passing extensive systemic exposure of drugs delivered to the lung, thus reducing side effects and administered doses.

So far, the greatest hurdle RNAi based therapy is facing is its safe and efficient delivery to organs other than the liver. Since siRNAs are negatively charged hydrophilic macromolecules, they cannot cross biological membranes by diffusion to reach their site of action, namely the cytoplasm of target cells. To protect the relatively labile, short siRNAs from enzymatic degradation, but also to mediate cellular uptake and release the nucleic acids inside the cells, a smart carrier system is therefore required.^[^ ^26^ ^]^ While delivery via attenuated viruses has been effective, it may elicit immune responses in the patient and bears the potential threat of recombining and regaining virulent properties.^[^ ^57^ ^]^ Thus, novel non‐viral delivery systems, such as polymeric nanoparticles or lipid‐based nanoparticles have gained importance.^[^ ^58^ ^]^

The lung offers the advantage of being an easily accessible organ for direct and localized administration of (siRNA)‐ therapeutics via intranasal or pulmonary administration. In contrast to systemic administration increasing the risk for potential siRNA degradation by serum proteins, direct pulmonary delivery of siRNA avoids interactions with serum which is absent on the mucosa of the lung.^[^ ^59^ ^]^

Bitko et al. were the first in 2004 to report that intranasal administration of siRNAs against RSV and PIV infections prevented and treated both single and concurrent infections in mice.^[^ ^60^ ^]^ They showed that prophylactic intranasal administration of 5 nmol (i.e., 70 µg) of anti‐RSV siRNA formulated with the transfection reagent, TransIT‐TKO prevented RSV infection. Similarly, using 5 nmol of the anti‐PIV siRNA eliminated PIV infection in mice. Interestingly, administration of “naked” unformulated siRNAs administered also resulted in substantial reduction of infection, which the authors estimated to be about 70–80% as effective as siRNA complexed with TransIT‐TKO. Using northern blot analysis, they showed that intranasal administration of siRNA results in accumulation in the lung, indicating that the viral inhibition was a specific consequence of the siRNAs targeting viral genes. They also showed no increase in type I or type II interferons in the lung. Around the same time, another study demonstrated that polyethyleneimine polyplexes containing siRNAs or plasmid DNAs encoding short hairpin RNAs (shRNA) against the influenza virus were able to inhibit virus production in the lung when given intravenously or intratracheally.^[^ ^61^ ^]^ The authors showed that the therapeutic effect was seen in both cases, when the siRNAs were administered before or after the infection, and the effect was found to be dose dependent. In a third parallel study in the same year, Tompkins and colleagues demonstrated in a mouse model of influenza, that siRNAs encapsulated in a lipid carrier administered first intravenously via hydrodynamic delivery 24 h before infection with the influenza virus, and then followed by a second siRNA dose intranasally after infection resulted in significant reduction in viral titers.^[^ ^62^ ^]^ These reports emphasize that appropriately designed siRNAs can be applied prophylactically or therapeutically by intranasal, intratracheal, or intravenously route of administration for protection from respiratory infection and as therapy after infection. Importantly, however, the results from Tompkins et al. must be taken with a pinch of salt since they used the non‐inflammatory siRNA control sequence against GFP as later pointed out by Robbins et al.^[^ ^53^ ^]^ In a murine infection model of influenza A, using non immunostimulatory 2′O‐methlylated siRNA and additional control siRNAs, Robbins et al. concluded that the in vivo anti‐influenza therapeutic effect of the sequence used by Tompkins et al. is primarily due to siRNA induced immune stimulation and is not mediated by sequence‐specific RNAi. These observations underline the importance of anticipating, monitoring, and adequately controlling siRNA‐mediated pro‐inflammatory effects for correct interpretation of therapeutic RNAi in vivo.

A notable example of antiviral RNAi is siRNA targeting RSV (ALN‐RSV01) which showed remarkable effects in humans targeting a highly conserved region in the mRNA coding for the RSV nucleocapsid protein.^[^ ^12^, ^63^ ^]^ ALN‐RSV01 was designed as a 19 bp RNA duplex with two (2′‐deoxy) thymidine overhangs on both 3′ ends that prevent its degradation by cellular nucleases. ALN‐RSV01 was tested as naked siRNA applied as nasal spray in adults that were experimentally infected with wild‐type RSV in a randomized, double‐blind, placebo‐controlled clinical trial. Patients were treated 2 days before and 3 days after RSV infection and ALN‐RSV01 was found to be safe, well tolerated and resulted in an overall 38% decrease in the number of infected individuals.^[^ ^63b^ ^]^ Furthermore, in another phase 2 randomized, double blind, placebo controlled clinical trial, ALN‐RSV01 showed a reduction in the risk of bronchiolitis obliterans syndrome in RSV‐infected lung transplant patients.^[^ ^64^ ^]^ However, after missing the primary endpoint in a phase 2b study, the reduction of progressive bronchiolitis obliterans syndrome in lung transplant patients, the program was abandoned in 2014. It has been speculated that it was potentially abandoned due to the emergence of drug resistant viruses, which is a common complication when only one siRNA molecule is used to target a single‐site of the viral genome. Hence, using several siRNAs targeting multiple genes within the RNA genome simultaneously seems to be a more promising approach.

Despite of the many advantages of local, pulmonary drug delivery, several hurdles have yet to be overcome for efficient therapeutic success. While siRNA delivery heavily relies on endocytosis of nano‐formulations, the presence of mucus, and the clearance of particles by macrophages but also cough and mucociliary clearance represent several of the metabolic barriers toward efficient pulmonary siRNA delivery.^[^ ^27^ ^]^ Additionally, the many bifurcations of the lung impose intrinsic anatomic barriers to inhalation medicine as discussed below. Nonetheless, as mentioned above, ALN‐RSV‐01 was successfully used as an intranasal “naked siRNA” against RSV. It can be speculated that the uptake of naked siRNA is, in part, due to the compromised membrane integrity of the virus‐infected pulmonary cells.^[^ ^60^ ^]^ It can, however, also be discussed that formulation of the siRNA in a nanocarrier may have resulted in better outcomes of the phase 2b study. And it needs to be considered that since these early days, striking advances have been made in the field including chemical modifications which make siRNA resistant to nucleases, enabling long‐lasting activities.^[^ ^65^ ^]^

Regardless of its mechanism, the use of naked siRNA eliminates toxicity concerns of the carriers, such as immune response, which is an important advantage in therapeutic applications.^[^ ^66^ ^]^ Moreover, naked siRNA lends itself easily to a simple inhaler‐based application as siRNA solutions can be aerosolized without integrity loss by various types of nebulizers.^[^ ^67^ ^]^ Nanoparticle suspensions of siRNA, on the other hand, are more prone to aggregation and other physical changes when exposed to shear forces during the aerosolization process.^[^ ^68^ ^]^ While chemically modified siRNAs have increased stability in vivo, they still cannot necessarily pass through intact cell membranes and may activate an immune response. Therefore, precise design of smart delivery systems is still a necessity for translating the potential of RNAi into therapeutic solutions. The different approaches to pulmonary antiviral siRNA delivery, route of administration and delivery vehicles used are summarized in **Table** **1** Aspects related to aerosol formulation and delivery are additionally discussed under 4.3.

**Table 1:** Examples of pulmonary antiviral siRNA delivery, route of administration, and delivery vehicles

Target virus	siRNA target	Delivery system	Experimental model	Administration	Reference
Respiratory syncytial virus	Fusion protein (F) Phosphoprotein (P)	OligofectAMINE reagent	Human lung cancer cell line, A549	‐	Bitko et al., 2001^[^ ^60^ ^]^
	Non‐structural protein 1 (NSP1) (Plasmid‐borne siRNA)	Chitosan nanoparticles	Mice infected with RSV	Intranasal	Zhang et al.,2005^[^ ^124^ ^]^
	P protein	Naked siRNA	BALB/c mice infected with RSV A2	Intranasal (2mg kg^−1^ body weight)	Zhang et al., 2008^[^ ^125^ ^]^
	N protein	Naked siRNA (ALN‐RSV01)	Phase II clinical trial	Aerosolized ALN‐RSV01 (0.6 mg/kg) by nebulization	DeVincenzo et al., 2010^[^ ^63b^ ^]^
Influenza	RNA dependent RNA polymerase (RdRP)	OligofectAMINE Reagent	Canine MDCK cell lines and embryonic chicken eggs	‐	Ge et al., 2003^[^ ^126^ ^]^
	Nucleocapsid protein (NP) Components of RNA transcriptase (PA and PB1)	PEI polyplexes	Influenza infection murine model	Intravenous	Ge et al., 2004^[^ ^61^ ^]^
	NP and PA proteins	OligofectAMINE reagent	BALB/c mice infected with influenza virus	Intranasal	Tompkins et al., 2004^[^ ^62^ ^]^
	Host gene, Ran‐binding protein 5 (*RANBP5)*	Lipofectamine 2000	Human 293T cells	‐	Deng et al., 2006^[^ ^41^ ^]^
SARS‐CoV	Spike protein (S)	Lipofectamine 2000	Vero E6 cells	‐	Wu et al., 2005^[^ ^127^ ^]^
	Spike protein (S) ORF1b (NSP12) regions	5% dextrose (D5W) solution	Rhesus Macaques	Intranasal	Li et al., 2005^[^ ^128^ ^]^
	Leader sequence (plasmid‐borne)	Calcium Phosphate transfection reagent	Human 293T cells Vero E6 cells	‐	Li et al., 2005 ^[^ ^129^ ^]^

**Table 2:** Chemical modifications of siRNA and their impact on stability, immunogenicity and in vivo behavior

Modification	Advantage	Note
Backbone modifications (phosphorothioate (PS) and phosphoester (PO) modifications)	Nuclease resistance, prolonged tissue retention.	PS modifications reduce binding affinity of oligo to its target and inhibit RNAi when in the center of the antisense strand.
Nucleobase modifications: 5‐ methylcytidine 5‐methyluridine	Increased melting temperature (*T*_m_ ) by 0.5 °C persubstitution.	
Abasic RNA	Decreased off target activity.	
Ribose sugar modifications: 2′O‐methyl (2′O‐Me)	Increased nuclease resistance at 5–30% 2′O‐Me modification (in vitro and in vivo), improved plasma stability.	Two or more consecutive 2′O‐Me inhibit RNAi. Stabilizes 3′‐endo ribose conformation. 2′O‐Me A, G, and U reduce immune response.
2′Fluoro (2′F)	Barely reduced RNAi if 2′F in all positions. Increased nuclease resistance at >50% modification.	2′F A reduces immune response.
2′O‐methoxyethyl (2′O‐MOE)	Stabilized 3′‐endo ribose conformation. Increased nuclease resistance with 2′‐MOE in terminal positions.	Replacement of the 9th or 10th nucleotide from the 5′‐end with 2′‐MOE increases probability of entry into RISC.
Locked nucleic acids (LNA)	Reduced conformational flexibility of nucleotides fixing the C3′‐endo conformation of the ribose. Increased nuclease resistance in vitro with ≥10–20% LNA.	>20% LNA in the antisense chain or the first LNA nt at 5′ end completely inhibit RNAi LNA can change the thermal asymmetry of the duplex, increasing siRNA efficiency

## 4 The SARS‐CoV‐2 Pandemic—A Case Study for RNAi Therapeutics

In December 2019, the World Health Organization (WHO) reported the outbreak of a novel coronavirus, the severe acute respiratory syndrome‐related coronavirus, designated as SARS‐CoV‐2 causing COVID‐19. The virus spread rapidly across 212 countries and as of December 11, 2020 the WHO reported more than 71 million confirmed cases of COVID‐19 worldwide, leading to the death of 1.6 million individuals and extraordinary social and economic disruption worldwide.^[^ ^69^ ^]^ So far, data suggest that the virus is manifold more severe than seasonal influenza and has a case fatality risk of about 1%, ranking it between the 1957 influenza pandemic (with a case fatality rate of 0.6%) and the 1918 influenza pandemic (2%).^[^ ^70^ ^]^ In contrast to the previous Middle East respiratory syndrome or severe acute respiratory syndrome (SARS) outbreaks, SARS‐CoV‐2 is transmitted efficiently even by people who themselves exhibit mild or no symptoms.^[^ ^71^ ^]^ SARS‐CoV‐2 is an enveloped, positive‐sense, single‐stranded RNA betacoronavirus of the family Coronaviridae.^[^ ^72^ ^]^ It has a 30 kb genome which encodes for 14 open reading frames (ORFs), including the surface glycoprotein, or spike (S) protein that is shared among all coronaviruses.^[^ ^73^ ^]^ The S protein is made up of two functional subunits, S1 and S2, which facilitate host‐virus interactions and viral entry.^[^ ^74^ ^]^ Cellular entry of SARS‐CoV(‐2) occurs via angiotensin‐converting enzyme 2 (ACE2),^[^ ^72^ ^]^ a type I transmembrane metallocarboxypeptidase. The physiologic function of ACE2 is negative regulation of the renin–angiotensin system, and it is therefore particularly expressed in the kidneys and lungs, but also in the gastrointestinal tract. For direct viral entry to the host cells, another host enzyme, the cellular serine protease TMPRS22, is also essential. While TMPRS22 is bound to ACE2, it can prime the S protein extracellularly for subsequent membrane fusion between the viral and plasma membranes and hence trigger viral entry to the host cells.^[^ ^75^ ^]^

Owing to its high expression of ACE2, the pulmonary system is particularly susceptible to SARS‐CoV‐2 infection. Following infection, four structural proteins, spike (S), envelope (E), membrane (M), and nucleocapsid (N), and other accessory proteins are expressed.^[^ ^72^ ^]^ Upon translation, the structural proteins are incorporated into the host endoplasmic reticulum to form virion precursors, which fuse with the genetic material and nucleocapsid in the cytoplasm and are exocytosed for infection of new cells. The host cells respond to the viral infection by excessive proinflammatory cell signaling resulting in increased secretion of inflammatory cytokines and chemokines, with a possible outcome of lung injury or acute respiratory distress syndrome (ARDS).^[^ ^76^ ^]^ However, due to the widespread expression of ACE2, complications also in other organs have been observed. Reports of neurological syndromes, clotting disorders and resulting stroke, kidney and liver failures have arisen as well as cases of “long‐COVID” where months after the acute infection several of these complications remain.

**Figure 2:**
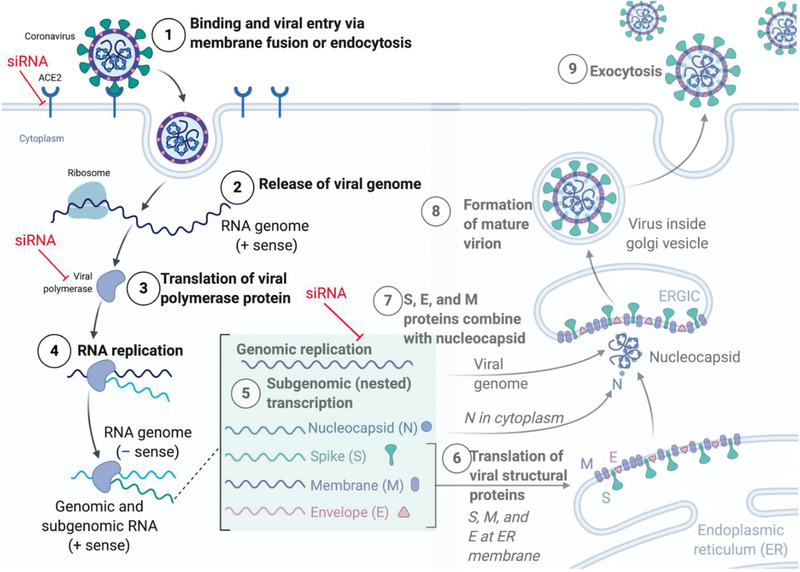
The SARS‐CoV‐2 infection lifecycle. After binding to the cellular ACE2 membrane receptor, SARS‐CoV‐2 is endocytosed and the viral genome released into the cytoplasm. Here, the polyprotein 1ab is translated directly from the (+) sense genome and cleaved into individual non‐structural proteins which form the replication transcription complex around the RNA genome. Now, full‐length genomic as well as subgenomic RNAs (sgRNA) are transcribed via negative sense RNA intermediates, and sgRNAs are subsequently translated into proteins, including the structural N, S, M and E proteins as well as ORF 3a, 6, 7a/b, and 8. Now the structural proteins assemble around full‐length RNA genomes to form progeny virus which is exocytosed. siRNA can be designed to inhibit the host–virus interactions by targeting host factors such as the ACE2 receptor, or viral genomic or subgenomic RNAs. Figure created with BioRender.com

While no small molecule or protein drugs have been available so far to attack SARS‐CoV‐2 specifically, many drugs were re‐purposed to combat COVID‐19, including antiviral (remedesivir, favipiravir, ritonavir),^[^ ^77^ ^]^ anti‐malarial (hydroxychloroquine),^[^ ^78^ ^]^ and anti‐cancer (interferon‐alpha 2b) agents.^[^ ^79^ ^]^ However, their efficacy against SARS‐CoV‐2 remains to be proven. In such situations where a novel virus emerges, the RNA based approaches, such as RNAi or mRNA provide a reliable and specific method to target the virus as soon as its genome is known. RNAi allows for addressing the root cause of the infection rather than palliating only the symptoms of the disease both in prophylactic or curative settings. Hence, labs across the world raced to sequence the SARS‐CoV‐2 genome and identified conserved regions that are essential for viral replication and survival for targeting by siRNAs and for the development of vaccines. Alternatively, host factors involved in the trafficking of the virus can in principle be silenced by siRNA. As compared to modulating host factors, such as ACE2, TMPRSS2, or the endocytic pathways involved in the internalization of the virus, targeting viral proteins using siRNAs is a safer, more direct and efficient approach. **Figure** **2** highlights the siRNA targeting possibilities for SARS‐CoV‐2 infections.

The four structural proteins encoded by the virus (S, M, E, and N) are all potential siRNA targets, since their sequences are largely conserved, and very few mutations can be tolerated in these regions.^[^ ^72^ ^]^ Of these four, the S glycoprotein has received the most attention and is the primary target for both vaccine and treatment development. The frontrunners in the SARS‐CoV‐2 vaccine development race, mRNA1273 by Moderna^[^ ^80^ ^]^ and BNT162b2 by BioNTech/Pfizer are lipid nanoparticle—encapsulated, nucleoside‐modified messenger RNA (mRNA)—based vaccines that encodes the SARS‐CoV‐2 (S) glycoprotein stabilized in its prefusion conformation. The S‐glycoprotein is a 150 kDa protein containing a fusion domain, a transmembrane domain, and a receptor binding domain (RBD) which interacts with the peptidase domain of ACE2.^[^ ^81^ ^]^ Targeting the S protein via RNAi would potentially reduce its levels within the cells, resulting in sub‐optimal assembly of viruses and could thus decrease its infectiousness.

Other than the S‐protein, the E and M structural proteins are also involved in the formation of the viral coat^[^ ^82^ ^]^ and are attractive targets for RNAi. The genome domain for the E‐protein is well‐conserved. In a study that investigated mutations in 68 samples of SARS‐CoV‐2, 42 missense mutations were detected in all the major structural and non‐structural proteins. However, no mutations were identified in the E‐protein.^[^ ^83^ ^]^ The E‐protein is a small, 8–12 kDa protein of essence for virus assembly and release. The M protein is found in the virion as a dimer and maintains the curvature of the viral membrane as well as its binding to nucleocapsids.^[^ ^84^ ^]^ The N‐protein is part of the nucleocapsid with each domain being able to bind to RNA^[^ ^85^ ^]^ via its phosphorylated residues. Each of these proteins is a potential target for RNAi therapy against SARS‐CoV‐2. However, after defining therapeutic targets for siRNA in respiratory viruses, their efficient delivery to relevant cells in the lung is the next hurdle.^[^ ^86^ ^]^

### 4.1 Pre‐Clinical Assessment of siRNA Therapeutics against Respiratory Viruses

Although direct delivery to the lung is possible, efficient intracellular delivery of siRNA in the lung at low toxicity is still a major challenge because of extra‐ and intracellular barriers the lung as an organ poses.^[^ ^27^ ^]^ Whereas administration of “naked” siRNA requires large doses to account for inefficient transport through mucus and cellular internalization of these negatively charged macromolecules, viral vectors can cause severe immunological responses, bear the risk of genome integration and long‐term side effects that would be undesired for the treatment of an acute infection. Additionally, they are mostly not specific for a certain target cell population. Targeted non‐viral siRNA carriers, on the other hand, offer several advantages.^[^ ^87^ ^]^ However, viruses have learnt to overcome barriers such as mucus in the conducting airways and surfactant in the alveolar region. Cationic lipid‐based siRNA nanocarriers, on the other hand, are easily destabilized by phospholipid‐containing surfactant which lines the lower airways.^[^ ^88^ ^]^ Cationic polymers can interact nonspecifically with respiratory mucus of the conducting airways and can thus get entrapped resulting in limited diffusion.^[^ ^88^ ^]^ To address this problem, shielded polymer nanocarriers that are decorated with poly(ethylene glycol) (PEG),^[^ ^56^, ^89^ ^]^ oligo(ethylene glycol),^[^ ^90^ ^]^ or the glycoprotein transferrin^[^ ^91^ ^]^ on their surface have been developed to decrease non‐specific interactions with mucus components for unhampered diffusion through the mucus layer.^[^ ^89c^ ^]^ Additionally, to improve targeting of specific cell types in the lung, siRNA nanocarriers have been modified with targeting ligands.^[^ ^91^ ^]^ Since the development of siRNA therapeutics requires screening of sequences and delivery approaches ideally in a model of infection, sophisticated in vitro models are required to determine their potential for therapeutic experiments in more expensive and time consuming in vivo models.

#### 4.1.1 Mimicking the Lung Environment

**Figure 3:**
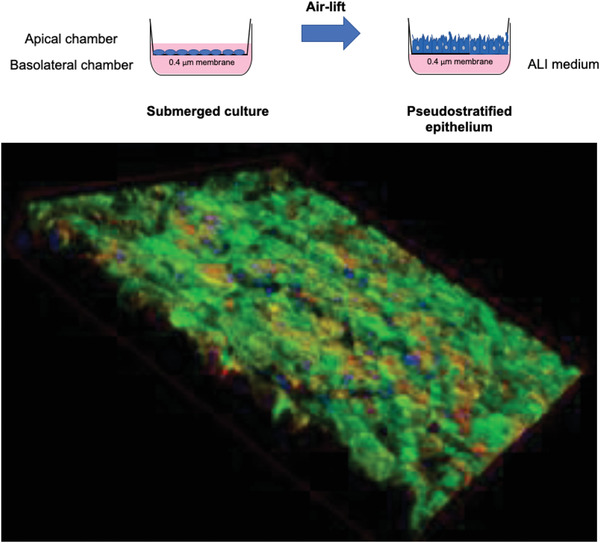
Schematic presentation of ALI cell culture and air‐lift (top) as well as an example of ALI‐cultured Calu‐3 cells (bottom, mucus stained with FITC‐wheat‐germ‐agglutinin (green), cytoskeleton (red), nuclei (blue)).

Considering that experimental infection of continuous cell lines does not take into account all aspects of viral pathogenesis,^[^ ^92^ ^]^ differentiated epithelial cells from airway tissue can be used to assess infections with airway viruses under more clinically relevant conditions. Accordingly, cell lines can be cultured under air–liquid interface (ALI) conditions and form a multilayered, polarized, and differentiated epithelium.^[^ ^93^ ^]^ This model is similar to the respiratory epithelium in terms of morphology and function, including mucus production and ciliary movement.^[^ ^94^ ^]^ For ALI culture, lung epithelial cells, such as A549, Calu‐3, or 16HBE, are seeded on porous membranes in so‐called transwells and cultured for 2 weeks in a medium‐covered state. The air‐lift then removes the medium from the apical chamber, leaving the epithelium to grow at the interface between the basolateral medium liquid and apical air (**Figure** **3** . ALI models are important and relevant in vitro cell culture models, especially for respiratory viruses that cannot multiply in 2D cell culture covered with liquid. Calu‐3 cells grown at ALI form dense cell layers with tight junctions as early as 2 days after air‐lift, as confirmed by transepithelial electrical resistance (TEER) values >1500 Ω cm^−2^. In addition, the cells secrete mucus as early as 2 days after air‐lift, as demonstrated by means of Wheat–Germ–Agglutinin staining (Figure 3 . ALI models with Calu‐3 cells, A549, and 16HBE cells are routinely used in models of respiratory virus infection,^[^ ^95^ ^]^ and most importantly, Calu‐3 cells were reported to express ACE2 apically,^[^ ^96^ ^]^ making for an ideal model to study SARS‐CoV‐2 infection.

#### 4.1.2 In Vivo Biodistribution and Efficacy

To obtain a better understanding of the bioavailable dose in the lung after pulmonary administration, biodistribution, and pharmacokinetic experiments need to be performed. Relevant dosing regimens can only be developed if local bioavailability is understood. Nuclear imaging approaches allow for quantitative and real time assessment of bioavailability and distribution processes.^[^ ^97^ ^]^ We reported earlier that larger doses reach the lung when siRNA nanocarrier formulations are instilled intratracheally as compared to intranasal administration^[^ ^27^ ^]^ and observed that nanoformulation can enhance lung retention significantly compared to siRNA administered as free nucleic acid.^[^ ^89a^ ^]^ Since intratracheal instillation, often used in animal models, bears the problem of administering comparatively large volumes of liquid as compared to the volume of the epithelium lining fluid, it is not a conducive administration route for clinical translation.^[^ ^98^ ^]^ However, intratracheal aerosolization with a microsprayer device such as the PennCentury Microsprayer^[^ ^99^ ^]^ in experimental animals so far is the best approach of mimicking efficient aerosol deposition in the lung after inhalation.

Additionally, we demonstrated that surface modification of siRNA nanocarriers has a strong impact on local versus systemic bioavailability after pulmonary administration.^[^ ^56^ ^]^ To put things into context, all of these experiments were performed in healthy animals. For relevant assessment of the potential of pulmonary delivery of siRNA against SARS‐CoV‐2, one needs to take into consideration that clearance mechanisms in the inflamed lung of patients with COVID‐19 showing neutrophilia^[^ ^100^ ^]^ significantly differ from physiological ones. Additionally, wild‐type mice cannot be infected with SARS‐CoV‐2 and require expression of human ACE2 such as the JAX Laboratories strain B6.Cg‐Tg(K18‐ACE2)2Prlmn/J.^[^ ^101^ ^]^ Several SARS‐CoV‐2 in vivo models are currently being developed including species other than mice.^[^ ^102^ ^]^ Understanding the biodistribution profile will be necessary to understand potential side effects in off‐target tissues. Additionally, dosing regimens can only be designed based on the lung retention of the administered siRNA formulations requiring knowledge on pharmacokinetics.

### 4.2 Pharmaceutical Development of RNAi Therapy of SARS‐CoV

The potential and promise of RNAi against COVID‐19 are also reflected in the efforts of the pharmaceutical industry. Within months of the initial outbreak and the publication of the SARS‐CoV‐2 genome, several companies, especially those with a focus on RNAi‐therapeutics, streamlined their efforts on the identification and development of siRNA sequences and formulations to prevent and treat SARS‐CoV‐2. Alnylam Pharmaceuticals for instance, partnered with Vir Biotechnology to jointly explore a library of siRNA sequences against the disease. In a press release, Alnylam reported the design of over 350 siRNA sequences targeting all currently available targets within the SARS‐CoV as well as SARS‐CoV‐2 genomes, to be screened in potency assays and subsequent anti‐viral activity assays in vitro and in vivo.^[^ ^103^ ^]^ As early as January 2020, almost 2 months into the start of the outbreak, Siranomics president and chief executive officer Patrick Lu announced that their research team had identified potent siRNA sequences to halt viral infection and replication. In a statement cited by NS Healthcare, Lu claimed that siRNA design, modification, and pulmonary delivery with a handheld nebulizer device combined could be the basis for development of siRNA‐based therapeutic and prophylactic strategies against SARS‐CoV‐2 infection.^[^ ^104^ ^]^ This statement is clearly based on Siranomics’ experience in the development of prophylactic and therapeutic siRNA‐based interventions for SARS coronavirus, H5N1 influenza, and other respiratory viral infections. Another important company is Suwan‐based OilX Pharmaceuticals Inc. where final preclinical work is claimed to be completed with the aim of clinical trials with their proprietary siRNA‐based COVID‐19 drug candidate.^[^ ^105^ ^]^ Their provisional patent, filed on 25th February 2020, covered more than 30 siRNA sequences against viral genes with the aim of preventing viral replication. These siRNA sequences could potentially also be extended to other coronaviruses, including MERS and SARS.

As mentioned, all three companies are aiming at pulmonary siRNA delivery. While the relative potencies and efficacy of the chosen siRNAs are yet to be published, the comparably short time before the initial outbreak of SARS‐CoV‐2 and the rapid development of target lead compounds reinforces the flexibility, potency, and specificity of RNAi to rapidly identify druggable targets. From the start of the outbreak, isolation, and genome sequencing of the novel SARS‐CoV‐2 virus to the development of initial testable siRNA sequences, it took a very short time of 3 months as compared to the regular pipeline of developing small molecule or protein‐based therapeutics. RNAi proves not only to be a specific, rapid, and potent approach to target virtually any protein in the human or viral genome but can also very quickly be used to identify and target genes in unknown genomes as in the case of emerging viruses. This advantage can result in rapid development of therapeutic options. Once the delivery system and formulation has been developed for intranasal or pulmonary administration, this modular approach also easily applies to different siRNA sequences in case of different viral targets or new virus outbreaks.

### 4.3 Aerosol Formulation Aspects

While Alnylam has experience with intranasal delivery,^[^ ^63b^ ^]^ Siranomics seems interested in the development of a formulation for a handheld nebulizer. As discussed above, intranasal administration can result in swallowing significant amounts of the dose.^[^ ^27^ ^]^ However, pulmonary inhalation requires the formulation of either a nebulizable liquid formulation or a dry powder. And both approaches are accompanied by hurdles and challenges.^[^ ^106^ ^]^ While in vivo studies in rodents can be performed using intratracheal aerosolization with one of three different devices, the PennCentury Microsprayer,^[^ ^99^ ^]^ the Micro‐Mist Nebulizer,^[^ ^107^ ^]^ and the AeroProbe nebulizing catheter,^[^ ^108^ ^]^ development of a product would require formulation development accordingly.

The pulmonary deposition of inhaled formulations depends on the aerodynamic diameter of their aerosol or powder particles and on the patient's pulmonary function.^[^ ^109^ ^]^ The optimal aerodynamic diameters for efficient delivery to the respiratory zones of the lung has been assumed to be between 1 and 5 µm.^[^ ^110^ ^]^ Particles with aerodynamic diameters larger than 6 µm can easily be deposited in the mouth and throat area due to impaction. Particles smaller than 1 µm have been reported to be exhaled again based on their diffusion within the exhaled breath or cleared by other clearance mechanisms.^[^ ^111^ ^]^ It needs to be considered, however, that nanoparticles, as also seen for particulate matter in air pollution,^[^ ^112^ ^]^ can indeed deposit in the lung.^[^ ^112^, ^113^ ^]^

Nebulization of a liquid formulation seems very straightforward, and aerosol and air jet nebulizers have been tested for nucleic acid aerosolization, with the former demonstrating higher performance. This observation was explained by lower shear forces due to the nominal exposure time to heat and shear force for nucleic acids.^[^ ^114^ ^]^ It is known that ultrasonic nebulization, in contrast, can alter aerosolized drug formulations, which is particularly unfavorable for sensitive macromolecules.^[^ ^115^ ^]^ However, liposomal carrier materials usually do not withstand the stresses in terms of temperature and shear force during nebulization, independent of the nebulizer type.^[^ ^116^ ^]^ Additionally, adhesion of positively charged particles to plastic parts of a nebulizer is possible, leading to preferential nebulization of the solute and severely limited siRNA emission through the mouthpiece.^[^ ^117^ ^]^ Therefore, after aerosolization, the biologic activity of the formulation has to be confirmed.^[^ ^108^, ^118^ ^]^

**Figure 4:**
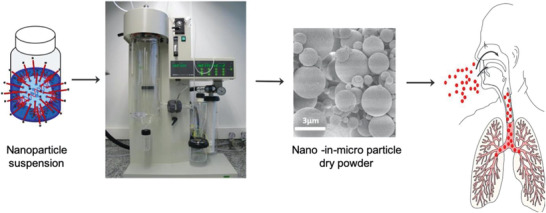
Schematic presentation of the spray drying process or siRNA nanocarriers into dry powder nano‐in‐micro formulations for inhalation. Reproduced with permission.^[^ ^123^ ^]^ Copyright 2019, Elsevier.

In contrast to nebulization, development of pressurized metered dose inhalers (pMDIs) and dry powder inhalation are other options for pulmonary drug delivery. While the development of pMDIs for nucleic acid nanoformulations is very tedious and sparsely described,^[^ ^119^ ^]^ we recently reviewed the literature describing different approaches of dry powder formulation of siRNA nanocarriers for pulmonary delivery.^[^ ^55^, ^120^ ^]^ While mainly free siRNA is formulated as dry powder,^[^ ^121^ ^]^ redispersion of nanocarriers after spray drying as well as the optimization of aerodynamic properties is often neglected (**Figure** **4** . For successful lung application, particle deposition near the target cells in the lungs is of fundamental importance and can be achieved with optimized particle size, shape, and density. By precisely controlling particle aerodynamic properties, certain regions in the lung can be reached.^[^ ^122^ ^]^ We recently developed a nanoparticle spray‐drying approach for efficient dry powder inhalation of plasmid DNA nanoparticles,^[^ ^123^ ^]^ and a platform technology has been developed for siRNA formulations.^[^ ^130^ ^]^

The additional advantage of a dry powder formulation is its extended shelf‐life. To develop a formulation that is suitable for all climate zones and to secure preparedness for future pandemic outbreaks, moisture content of the powder after extended storage in different climate conditions needs to monitored. Efficacy after spray drying and after storage has to be confirmed as well, and preclinical efficacy in rodents can be determined using a powder insufflator.

## 5 Future Outlook

Respiratory viral infections are a severe public health threat with significant personal, social, and economic consequences, as seen with the current SARS‐CoV‐2 pandemic. However, despite an urgent need, by the beginning of the pandemic, hardly any effective therapeutic or prophylactic treatments were available. RNAi has shown to be a promising strategy to control viral infections by silencing viral or cellular proteins that are essential for viral replication. Since RNAi depends on the Watson Crick base pairing between the siRNA and the target mRNA, it theoretically allows to target any gene in a specific manner. However, the combination of small genome size and a relatively high mutation rate, poses difficulties for developing RNAi‐based therapies especially for RNA viruses. In this regard, recent advancements in bioinformatics and genome sequencing technologies greatly facilitate the identification, and in silico assessment allows for better evaluation of off target interactions. Moreover, over the last years, RNAi has been used in large‐scale, genome‐wide screens in cell lines, which also make it possible to understand genes required for host‐virus interactions and to identify host genes that can be targeted against viral infections.

Besides the identification of target sequences and siRNA design, the delivery and stability of siRNAs remains one of the biggest challenges to siRNA therapies. Naked siRNAs are very sensitive to nuclease degradation in the extracellular space and can induce the innate immune system. To increase their stability and decrease their immunogenicity, siRNAs can be chemically modified by changes to their oligo backbone, by incorporating nucleotides analogs or modifying the ribose sugar. As seen in the work from Robbins et al, the immunogenicity of siRNAs, both control as well as the targeting siRNA, plays a critical role in their evaluation as therapeutic molecules, especially as antivirals.^[^ ^53^ ^]^ Simultaneously, scientists are working tirelessly to develop siRNA carrier molecules, either as lipid nanoparticles, or polymer‐based delivery vehicles or as oligos conjugated to the targeting ligand for the delivery of siRNAs to target cells.

Due to the extensive first‐pass effect of nano materials after systemic delivery, local administration routes for delivery of siRNA nanoformulations is a promising alternative, particularly for treating diseases outside the liver. To target the pulmonary epithelium, siRNAs can be inhaled which is a non‐invasive, convenient method to achieve high local siRNA concentrations in the lung such that sufficient quantities can reach infected epithelial cells. Importantly, siRNA can also be administered prophylactically to prevent an infection.

## 6 Conclusion

Taken together, inhaled siRNA holds great promise to develop antiviral therapies for respiratory diseases. Once a pipeline of siRNA design and delivery to the respiratory tract is established, the adaptation of RNAi‐based therapies to new targets is comparably simple. Thus, it could allow a relatively fast development of antiviral drugs in emergency settings caused by newly emerging pathogens affecting the respiratory tract such as SARS‐CoV‐2.

## Conflict of Interest

The authors declare no conflict of interest.
